# Incidence of memory complaints during the COVID-19 pandemic in Southern Brazil: findings from PAMPA cohort

**DOI:** 10.1590/1980-5764-DN-2022-0072

**Published:** 2023-05-05

**Authors:** Natan Feter, Eduardo Lucia Caputo, Jayne Santos Leite, Felipe Mendes Delpino, Júlia Cassuriaga, Carine Nascimento da Silva, Marcelo Cozzensa da Silva, Felipe Fossati Reichert, Airton José Rombaldi

**Affiliations:** 1 Universidade Federal de Pelotas, Escola Superior de Educação Física, Programa de Pós-Graduação em Educação Física, Pelotas RS, Brazil.; 2 Universidade Federal do Rio Grande do Sul, Faculdade de Medicina, Programa de Pós-Graduação em Epidemiologia, Porto Alegre RS, Brazil.; 3 Universidade Federal do Rio Grande do Sul, Faculdade de Medicina, Programa de Pós-Graduação em Ciências da Saúde, Porto Alegre RS, Brazil.; 4 Universidade Federal de Pelotas, Faculdade de Enfermagem, Programa de Pós-Graduação em Enfermagem, Pelotas RS, Brazil.; 5 Universidade Federal do Rio Grande, Faculdade de Medicina, Programa de Pós-Graduação em Ciências da Saúde, Rio Grande RS, Brazil.

**Keywords:** COVID-19, Mental Health, Memory, Cognitive Dysfunction, COVID-19, Saúde Mental, Memória, Disfunção Cognitiva

## Abstract

Population-level studies investigating the incidence of memory complaints during the COVID-19 pandemic are scarce.

**Objective:**

This study aimed to examine the incidence of memory complaints over 15 months during the COVID-19 pandemic in adults from Southern Brazil.

**Methods:**

Data from the PAMPA (Prospective Study about Mental and Physical Health in Adults) cohort, a longitudinal study with adults residing in Southern Brazil, were analyzed. An online-based, self-administered questionnaire was used to assess self-rated memory. Participants rated their memories as excellent, very good, good, fair, or poor. Incident memory complaints were defined as worse memory perception from baseline to follow-up. Cox proportional hazard models were used to identify factors associated with the increased risk of memory complaints.

**Results:**

During follow-up, a cumulative incidence of 57.6% for memory complaints was observed. Female sex (hazard ratio [HR] 1.49; 95% confidence intervals [CI] 1.16–1.94), lack of access to prescribed medicine (HR: 1.54; 95%CI 1.06–2.23), and worsened anxiety symptoms (HR: 1.81; 95%CI 1.49–2.21) were associated with an increased risk of memory complaints. Regular practice of physical activity was associated with a reduced risk of memory complaints (HR: 0.65; 95%CI 0.57–0.74).

**Conclusion:**

Since the COVID-19 pandemic, 6 in 10 adults in Southern Brazil have developed memory complaints. Factors including sex and lack of medications increased the risk of incident memory complaints. Physical activity reduced the risk of incident memory complaints during the COVID-19 pandemic.

## RESUMO.

Estudos em nível populacional que investiguem a incidência de queixa na memória durante a pandemia de COVID-19 são escassos.

**Objetivo:**

Nosso objetivo foi examinar a incidência de queixa na memória, ao longo de 15 meses durante a pandemia de COVID-19, em adultos do Sul do Brasil.

**Métodos:**

Foram analisados dados da coorte Estudo Prospectivo sobre Saúde Mental e Física em Adultos (PAMPA), um estudo longitudinal com adultos residentes no Sul do Brasil. Um questionário autoadministrado *online* foi usado para avaliar a memória autorreferida. Os participantes classificaram sua memória como excelente, muito boa, boa, regular ou ruim. A queixa na memória incidente foi definida como pior percepção de memória desde a linha de base até o acompanhamento. Modelos de risco proporcional de Cox foram usados para identificar fatores associados ao aumento do risco de queixa na memória incidente.

**Resultados:**

Durante o seguimento, observou-se incidência cumulativa de 57,6% para queixa na memória. Sexo feminino (*hazard ratio* — HR 1,49; intervalo de confiança — IC 95% 1,16–1,94), falta de acesso ao medicamento prescrito (HR: 1,54; IC95% 1,06–2,23) e sintomas de ansiedade (HR: 1,81; IC95% 1,49–2,21) foram associados a risco aumentado de queixa na memória incidente. A prática regular de atividade física foi vista como fator protetor (HR: 0,65; IC95% 0,57–0,74).

**Conclusões:**

Desde a pandemia de COVID-19, seis em cada dez adultos no Sul do Brasil desenvolveram queixa na memória. Fatores como sexo e falta de medicamentos aumentaram o risco de queixa na memória incidente. A atividade física reduziu o risco de queixa na memória incidente durante a pandemia de COVID-19.

## INTRODUCTION

The COVID-19 pandemic has triggered numerous consequences to global public health. Healthcare professionals and scientists raised concerns about the potentially adverse effects of COVID-19-induced social distancing on psychiatric and neurological functions, including memory complaints^1–3^. Previous studies have shown memory concerns as a common neurological symptom in diagnosed^3^ and non-diagnosed individuals^4^.

Memory complaints, defined as self-perceived worsening in memory, are a potential indicator of the early stages of cognitive impairment and dementia^5–7^. It is also associated with impaired objectively measured cognitive function^8,9^, white matter lesion^8^, and hippocampal volume^8^. In large epidemiological studies, assessing memory complaints may provide valuable information on brain functioning. Memory problems are closely associated with depression^10^. Although a causal association between these two conditions is unclear, an increase in the incidence of memory complaints as an indirect consequence of the COVID-19 pandemic may be expected. However, population-level studies investigating the incidence of memory complaints during the COVID-19 pandemic are scarce. We investigated the incidence and factors associated with memory complaints over 15 months during the COVID-19 pandemic in adults from Southern Brazil.

## METHODS

### Study design

The present study analyzed data from the PAMPA (Prospective Study about Mental and Physical Health in Adults) cohort, an ongoing, population-based, longitudinal study with adults living in the Rio Grande do Sul state, Brazil. Baseline assessment occurred during wave 1 (June and July 2020). Two consecutive waves were completed 6 months apart (wave 2: December 2020 and January 2021; wave 3: June and July 2021). More details about the study design, sampling process, and data collection can be found elsewhere^11^. All participants provided formal consent. The study protocol was approved by the institutional research ethics board of the Universidade Federal de Pelotas, Brazil (protocol: 4.093.170). Adults (≥18 years) living in the Rio Grande do Sul state were eligible for the study. To reach our target sample, a four-arm recruitment strategy was used based on local media, professional colleagues’ contacts, social media, and city- and state-level health offices. The recruitment phase lasted 2 months in every survey. Participants filled out an online-based, self-administered questionnaire in each survey.

### Incident memory complaints

Participants were asked to rate their current memory in the three waves using the same question: “How do you rate your memory today?” In wave 1, participants were also asked to evaluate their memory before the COVID-19 pandemic using the following question: “How do you rate your memory before social distancing?” The options for both questions were “excellent,” “very good,” “good,” “fair,” or “poor.” Incident memory complaints were defined as worse memory perception from baseline to follow-up. The time event was defined as the first wave of participants who were classified as having memory complaints. Similar questions have been used in previous epidemiological studies^12,13^.

### Exposures

Demographic questions included information about age, sex, ethnicity, educational achievement, and marital status. We included questions on aspects related to the COVID-19 pandemic, such as daily routines during social distancing and reduced monthly income since the pandemic started. Self-reported body weight, height, and whether there had been a clinical diagnosis of chronic disease (e.g., hypertension, diabetes, and depression) were asked using the questions from the Brazilian Surveillance System of Risk Factors for Chronic Diseases by Telephone Interviews^14^. Data of the four most frequently reported conditions were shown, with the remaining merged as “others.” Physical activity was assessed using a validated single-item question about the amount of physical activity practiced in the last 7 days before the survey^15^. The volume of physical activity was calculated as minutes per week and further categorized as inactive (<150 min per week) and active (≥150 min per week) according to the World Health Organization 2020 guidelines on physical activity^16^. The Hospital Anxiety and Depression Scale was used to identify changes in anxiety and depression symptoms from pre-pandemic (retrospectively) and wave 1 (last week as reference). Participants were classified as having worse, better, or unchanged symptoms if they scored higher, lower, or the same from before to during the pandemic, respectively.

### Statistical analysis

Data were reported as proportions and respective 95% confidence intervals (CIs). Chi-square and linear trend tests were used for categorical variables. Cox proportional hazard models were used to identify the factors associated with the risk of incident memory complaints in the cohort. Model 1 was a crude analysis. Variables that reach the cutoff value of p≤0.20 in the crude analysis were included in the multivariable models 2–5. Model 2 included sociodemographic covariates. Model 3 included variables from the previous model plus COVID-19-related covariates. Model 4 included model 3 plus preexistent chronic conditions and psychiatric symptoms. Model 5 included model 4 plus physical activity. All analyses were performed using Stata 14.2 (StataCorp, College Station, TX, USA). Subjective sensitivity analysis excluded participants who reported a positive test for COVID-19 from waves 1 to 3 (n=608). Results were expressed as hazard ratio (HR), incidence rate (IR), and respective 95%CI.

## RESULTS

**Figure 1:**
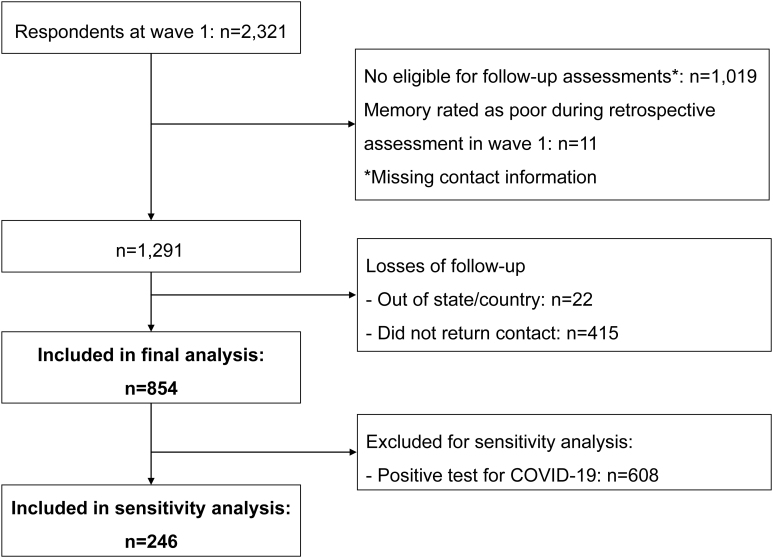
Study workflow.

A total of 854 participants (Figure 1) who self-rated their memory during baseline were followed up over the first 15 months of the COVID-19 pandemic (March 2020 to June 2021). These participants were mostly women, aged between 31 and 59 years, with an academic degree, and white. During the first 3 months of the COVID-19 pandemic, two in five people had decreased monthly income, and about two-thirds reported that they left home only to do essential activities. One in five reported a diagnosis of depression, while one in ten had hypertension or high blood cholesterol. Three-quarters of respondents were physically inactive.

During follow-up, 362 individuals reported having memory complaints, which accounts for a cumulative incidence of 57.6% (95%CI: 54.3%-60.9%). Women aged 30 years or younger and who had reduced monthly income presented a higher risk of incident memory complaints (Table 1). The daily routine during the pandemic was not associated with the incidence of memory complaints. On the contrary, those living with depression, lacking access to prescribed medicine during the pandemic, and being physically inactive showed a higher risk of such a problem.

**Table 1:** Sociodemographic, behavioral, and health-related characteristics of the sample. Rio Grande do Sul, Brazil (n=854).

		Total sample (n=854)	Incident memory complaint		p-value
			No (n=362)	Yes (n=492)	
**Demographic**					
Sex					
	Male	21.4 (18.7–24.2)	56.0 (48.7–63.1)	44.0 (36.9–51.3)	<0.001*
	Female	78.6 (75.8–81.3)	38.7 (35.0–42.4)	61.3 (57.6–65.0)	
Age, years					
	30 or younger	38.9 (35.7–42.2)	35.8 (30.9–41.2)	64.2 (58.8–69.1)	<0.001†
	31–59	52.5 (49.1–55.8)	44.9 (40.3–49.5)	55.1 (50.5–59.7)	
	60 or older	8.7 (7.0–10.8)	56.8 (45.2–67.6)	43.2 (32.4–54.8)	
Educational achievement					
	High school or lower	30.0 (27.0–33.1)	39.5 (33.6–45.6)	60.5 (54.4–66.4)	0.256*
	University degree or higher	70.0 (66.9–73.0)	43.6 (39.7–47.7)	56.4 (52.3–60.3)	
Skin color					
	White	91.1 (89.0–92.8)	41.7 (38.3–45.2)	58.3 (54.8–61.7)	0.239*
	Mixed	5.2 (3.9–6.9)	47.7 (33.4–62.4)	52.3 (37.5–66.6)	
	Black	3.4 (2.4–4.9)	48.3 (30.7–66.2)	51.7 (33.8–69.3)	
	Other‡	0.4 (0.1–1.1)	n=2	n=1	
Conjugal status					
	With partner	60.4 (57.1–63.7)	44.6 (40.3–48.9)	55.4 (51.1–59.7)	0.110*
	Living alone	39.6 (36.3–42.9)	39.1 (34.0–44.4)	60.9 (55.6–66.0)	
**COVID-19 pandemic-related**					
Reduced monthly income					
	No	55.9 (52.5–59.2)	46.5 (42.1–51.0)	53.5 (49.0–57.9)	0.006*
	Yes	44.1 (40.8–47.5)	37.1 (32.4–42.1)	62.9 (57.9–67.6)	
Daily routine					
	At home most of the time	6.6 (5.1–8.4)	35.7 (24.2–49.1)	64.3 (50.9–75.8)	0.407†
	Left home only to essentials	66.7 (63.5–69.8)	41.9 (37.9–46.0)	58.1 (54.0–62.1)	
	Left home all days	26.7 (23.8–29.8)	45.2 (38.8–51.7)	54.8 (48.3–61.2)	
**Chronic conditions**					
Hypertension					
	No	86.1 (83.6–88.2)	42.4 (38.9–46.1)	57.6 (53.9–61.1)	0.929*
	Yes	13.9 (11.8–16.4)	42.0 (33.4–51.1)	58.0 (48.9–66.6)	
Diabetes					
	No	96.1 (94.6–97.2)	42.8 (39.4–46.2)	57.2 (53.8–60.6)	0.283*
	Yes	3.9 (2.8–5.4)	33.3 (19.3–51.1)	66.7 (48.9–80.7)	
Depression					
	No	79.7 (76.9–82.3)	44.3 (40.6–48.1)	55.7 (51.9–59.4)	0.002*
	Yes	20.3 (17.7–23.1)	34.7 (27.9–42.1)	65.3 (57.9–72.1)	
High cholesterol					
	No	88.6 (86.3–90.6)	41.9 (38.4–45.4)	58.1 (54.6–61.6)	0.397*
	Yes	11.4 (9.4–13.7)	46.4 (36.7–56.4)	53.6 (43.6–63.3)	
Other					
	No	78.9 (76.1–81.5)	42.6 (38.9–46.4)	57.4 (53.6–61.1)	0.825*
	Yes	21.1 (18.5–23.9)	41.7 (34.7–49.0)	58.3 (51.0–65.3)	
**Psychiatric symptoms**					
Worsened anxiety symptoms					
	No	51.1 (47.7–54.4)	66.3 (61.2–71.0)	39.8 (35.6–44.2)	<0.001*
	Yes	49.9 (45.6–52.3)	33.7 (29.0–38.7)	60.2 (55.7–64.4)	
Worsened depressive symptoms					
	No	69.3 (65.8–72.5)	74.9 (69.9–79.2)	64.4 (59.5–69.1)	0.002*
	Yes	30.7 (27.5–34.2)	25.1 (20.8–30.1)	35.6 (30.9–40.5)	
Lack of access to prescribed medicine during the pandemic					
	No	27.8 (24.8–30.9)	44.7 (38.5–51.1)	55.3 (48.9–61.5)	0.010*
	Yes	6.2 (4.8–8.0)	22.6 (13.2–36.0)	77.4 (64.0–86.8)	
	No regular use of medicine		43.3 (39.2–47.4)	56.7 (52.6–60.8)	
**Nutritional**					
Body mass index					
	Normal	44.9 (41.6–48.3)	39.3 (34.5–44.3)	60.7 (55.7–65.5)	0.269†
	Overweight	34.9 (31.8–38.2)	43.8 (38.2–49.5)	56.2 (50.5–61.8)	
	Obese	20.2 (17.6–23.1)	45.9 (38.6–53.4)	54.1 (46.6–61.4)	
**Behavioral**					
Physical activity at baseline					
	Inactive	73.8 (70.7–76.6)	39.2 (35.5–43.1)	60.8 (56.9–64.5)	0.002*
	Active	26.2 (23.4–29.3)	51.3 (44.8–57.8)	48.7 (42.2–55.2)	

Notes:

* Chi-square test [% (95%CI)] to compare the difference in the proportion of participants among groups;

† Linear trend test.

‡ Insufficient number of observations to calculate 95%CI.

**Figure 2:**
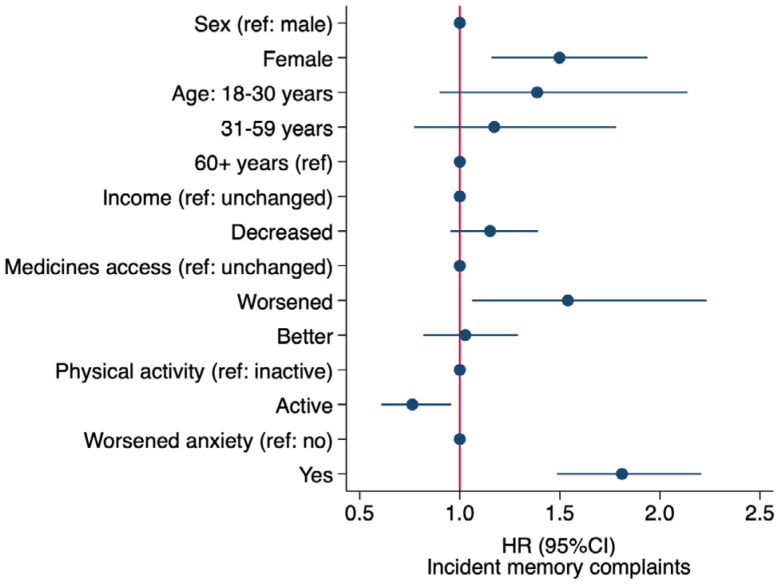
Factors associated with incident memory complaints during the COVID-19 pandemic in Southern Brazil (n=854).

Factors associated with the risk of incident memory complaints are illustrated in Figure 2 and described in Table 2. Female sex and age 30 or lower were associated with an increased risk of incident memory complaints, although the association with age was lost after including changes in psychiatric symptoms in the model. The IR in women (141.4; 95%CI 127.7–156.5; p<0.001) and in participants aged 30 years or younger (IR: 155.1; 95%CI 124.9–178.4; p=0.009) was significantly higher than men (88.9; 95%CI 70.5–112.0) and adults aged 60 years or older (79.7; 95%CI 54.3–117.1). Loss of monthly income was also associated with increased IR (149.3; 95%CI 130.6–170.7; p=0.005) compared to participants whose monthly income was not affected by the pandemic (IR: 114.6; 95%CI 100.7–130.3). Participants who reported a lack of access to prescribed medicine during the pandemic had higher IR of incident memory complaints (212.0; 95%CI 154.9–290.1; p=0.002) than those whose access was not impaired (115.8; 95%CI 96.3–139.2). However, the inclusion of other covariates in the model did not change the HR for incident memory complaints or monthly income. Physically active adults had lower HR and IR of memory complaints (101.6; 95%CI 83.6–123.5; p=0.004) than inactive peers (140.1; 95%CI 126.1–155.7).

**Table 2:** Incidence rate of memory complaint during the COVID-19 in adults at Southern Brazil (n=854).

		Model 1 HR (95%CI)*	p-value	Model 2 HR (95%CI)†	p-value	Model 3 HR (95%CI)†	p-value	Model 4 HR (95%CI)†	p-value	Model 5 HR (95%CI)†	p-value
**Demographic**											
Sex											
	Male (ref)	**1.00**	**0.002** *	**1.00**	**0.003** *	**1.00**	**0.002** *	**1.00**	**0.006** *	**1.00**	**0.002**
	Female	**1.58 (1.24–2.00)**		**1.50 (1.16–1.93)**		**1.49 (1.16–1.93)**		**1.49 (1.12–1.97)**		**1.49 (1.16–1.94)**	
Age, years											
	30 or younger	**1.84 (1.22–2.77)**	**0.001†**	**1.70 (1.13–2.57)**	**0.004†**	**1.60 (1.05–2.46)**	**0.017†**	1.27 (0.81–1.99)	0.069†	1.39 (0.90–2.14)	0.051†
	31–59	**1.45 (0.97–2.18)**		**1.39 (0.93–2.09)**		**1.35 (0.89–2.04)**		1.02 (0.66–1.57)		1.17 (0.77–1.78)	
	60 or older (ref)	**1.00**		**1.00**		**1.00**		1.00		1.00	
Educational achievement											
	High school or lower (ref)	1.00	0.278*								
	University degree or higher	0.90 (0.75–1.09)									
Skin color											
	White (ref)	1.00	0.802*								
	Black	0.87 (0.57–1.32)									
	Mixed	0.89 (0.53–1.48)									
	Other	0.54 (0.08–3.87)									
Conjugal status											
	Living alone (ref)	1.00	0.113	1.00	0.389*						
	With partner	1.16 (0.97–1.38)		1.09 (0.90–1.33)							
**COVID-19 pandemic-related**											
Reduced monthly income											
	No (ref)	1.00	**0.002** *			**1.00**	**0.038** *	1.00	0.149*	1.00	0.144*
	Yes	**1.23 (1.03–1.47)**				**1.22 (1.01–1.47)**		1.15 (0.95–1.39)		1.15 (0.95–1.39)	
Daily routine											
	At home most of the time (ref)	1.00	0.541*								
	Left home only to essentials	0.88 (0.62–1.24)									
	Left home all days	0.82 (0.56–1.18)									
Lack of access to prescribed medicine during the pandemic											
	No	1.00	**0.010** *			**1.00**	**0.037** *	**1.00**	**0.037** *	**1.00**	**0.023** *
	Yes	**1.73 (1.20–2.49)**				**1.54 (1.06–2.23)**		**1.55 (1.07–2.25)**		**1.54 (1.06–2.23)**	
	No regular use of medicine	1.10 (0.89–1.37)				1.00 (0.79–1.25)		1.00 (0.80–1.26)		1.03 (0.82–1.29)	
**Chronic conditions**											
Hypertension											
	No	1.00	0.864*								
	Yes	1.02 (0.79–1.33)									
Diabetes											
	No	1.00	0.223*								
	Yes	1.31 (0.85–2.00)									
Depression											
	No	**1.00**	**0.015** *					1.00	0.250*		
	Yes	**1.31 (1.06–1.64)**						1.16 (0.90–1.50)			
High cholesterol											
	No	1.00	0.596*								
	Yes	0.92 (0.68–1.24)									
Other											
	No	1.00	0.777*								
	Yes	0.97 (0.76–1.22)									
**Psychiatric symptoms**											
Worsened anxiety symptoms											
	No	**1.00**	**<0.001** *					**1.00**	**<0.001** *	**1.00**	**<0.001** *
	Yes	**1.97 (1.62–2.38)**						**1.84 (1.51–2.25)**		**1.81 (1.49–2.21)**	
Worsened depressive symptoms											
	No	**1.00**	**<0.001** *					1.00	0.315*		
	Yes	**1.41 (1.13–1.75)**						1.13 (0.89–1.43)			
**Nutritional**											
Body mass index											
	Normal	1.00	0.241†								
	Overweight	0.90 (0.73–1.09)									
	Obese	0.88 (0.69–1.12)									
**Behavioral**											
Physical activity at baseline											
	Inactive	1.00	**0.009** *							**1.00**	**0.018** †
	Active	0.75 (0.60–0.93)								**0.65 (0.57–0.74)**	

Notes: Bold values indicate p<0.05;

* p<0.05 compared to the reference group;

† p for linear trend.

Model 1: Crude; Model 2: Age, sex, conjugal; and Model 3: Model 2 plus reduced monthly income and lack of access to prescribed medicine; Model 4: Model 3 plus change in depression and anxiety symptoms and clinically diagnosed depression; Model 5: Model 4 plus physical activity.

**Table 3:** Incidence rate of memory complaint in adults at Southern Brazil excluding participants whose tested positive for COVID-19 during follow-up (n=246).

		HR (95%CI)	p-value
**Demographic**			
Sex			
	Male (ref)	1.00	0.170
	Female	1.25 (0.91–1.72)	
Age, years			
	30 or younger	1.44 (0.82–2.52)	0.163
	31–59	1.27 (0.74–2.19)	
	60 or older (ref)	1.00	
**COVID-19 pandemic-related**			
Reduced monthly income			
	No (ref)	1.00	0.192
	Yes	1.17 (0.92–1.50)	
Lack of access to prescribed medicine during the pandemic			
	No	**1.00**	**0.095**
	Yes	**1.88 (1.20–2.94)**	
	No regular use of medicine	1.08 (0.81–1.45)	
**Psychiatric symptoms**			
Worsened anxiety symptoms			
	No	**1.00**	**0.031**
	Yes	**1.63 (1.05–2.54)**	
**Behavioral**			
Physical activity at baseline			
	Inactive	1.00	**0.034**
	Active	**0.72 (0.54–0.98)**	

Notes: Bold values indicate p<0.05. Adjusted for age, sex, reduced monthly income, changes in anxiety symptoms, and physical activity.

The associations between depressive symptoms and clinically diagnosed depression with memory complaints were lost when the model included other covariates. Mediation and interaction analyses were performed; however, nonsignificant associations were found (p>0.10). Therefore, our data indicate that the expected association between depression and memory complaints may be driven by other factors, including sex, access to medicine, changes in anxiety symptoms, and physical activity. In the sensitivity analysis (Table 3), only the lack of medication, changes in anxiety symptoms, and physical activity remained associated with the HR of memory complaints.

## DISCUSSION

To the best of our knowledge, this is the first study that examined the incidence of memory complaints during the COVID-19 pandemic in Brazil. We showed that between March 2020 and July 2021, 57.6% of the participants reported memory complaints during the 15-month follow-up. Female sex, age 30 years or lower, reduced monthly income, and a lack of access to prescribed medicine were associated with an increased risk of incident memory complaints. On the contrary, physical activity was identified as a protective factor against memory complaints.

Since the start of the COVID-19 pandemic, many studies have emerged, especially based on cross-sectional design and focusing on the deleterious impact of the pandemic on mental health^17^. As the pandemic progressed, longitudinal studies were carried out, and a reduction in depressive and anxiety symptoms was observed^18^. However, other psychiatric symptoms and cognitive impairment should be investigated as a long-term consequence of the pandemic for both COVID-19-diagnosed and non-diagnosed individuals. Recent studies have documented a high frequency of neuropsychiatric manifestations after COVID-19, including cognitive dysfunction^19^. A meta-analysis examining the persistent symptoms after COVID-19 illness, also known as long COVID, pooled data from 41 cross-sectional studies and 1,680,003 individuals who tested positive for COVID-19^3^. The study reported that one in seven participants reported memory issues after COVID-19 infection. However, data on the neuropsychiatric consequences of the COVID-19 pandemic on non-diagnosed individuals are scanty. A previous report from the PAMPA cohort showed that 3 months after the COVID-19 pandemic was declared, one in three participants reported a decline in memory functioning compared to the pre-pandemic period^4^. In the present study, we showed that roughly three in five adults residing in Southern Brazil noticed a decline in memory functioning since the start of the COVID-19 pandemic.

The incidence of memory complaints may increase in part because of the increased frequency of elevated depressive symptoms^10^. However, data from the present study rejected such a hypothesis, as depression was not associated with incident memory complaints in the final model. We identified female sex, young age, reduced monthly income, lack of access to prescribed medications, and physical inactivity as factors associated with memory complaints. Besides the low number of participants aged 60 years or older, we hypothesized that young adults were more likely to suffer undesirable consequences of social distancing measures, including job losses, which led to reduced income. Similarly, the psychological burden of the pandemic and its impact on work-family activities that conflict with healthy behaviors were greater in women, especially at the early stages of the pandemic^1,20^. In addition, a lack of prescribed medications may lead to unstable cardiovascular and metabolic conditions, eventually, affecting brain functions. Therefore, we hypothesized that the burden of the COVID-19 pandemic on the abovementioned groups may have led to neuropsychological disturbances independently of preexisting chronic conditions and COVID-19 infection.

Our study also identified a protective association between physical activity and the risk of incident memory complaints. Previous studies have confirmed the protective benefits of physical activity on the risk of cognitive impairment and dementia^21,22^. Although beyond the scope of the present study, exercise-induced neurogenic mechanisms including the release of brain-derived neurotrophic factors into the bloodstream are strongly associated with improved memory functioning^23,24^. However, reports from the early phases of the pandemic revealed a decrease in physical activity practice^25^. Such a decline may be followed by an insufficient increase in physical activity^26,27^. Hence, surveillance of physical activity is required to examine its association with the incidence of long-term consequences of the COVID-19 pandemic, including memory complaints.

The present study provides novel information on the neuropsychological consequences of the COVID-19 pandemic in the adult population of Southern Brazil. Nevertheless, some limitations must be acknowledged. First, we were not able to objectively assess cognitive function in the cohort as face-to-face interviews were not allowed by the local ethics board in response to the COVID-19 pandemic. Second, the memory from the pre-pandemic period was assessed retrospectively. Although recall bias cannot be ruled out, the COVID-19 pandemic is such an unprecedented event that it impacted everyone, so, there is no clear reason why some people would remember differently than others regarding factors such as behavioral habits or health issues. Third, our sample is overrepresented by respondents with one or more academic degrees. This sampling bias was expected since data collection was online, and poorer/less educated individuals have less access to the Internet compared to richer/more educated people in Brazil. However, COVID-19 is likely to have had a larger impact on the lower economic groups of the population; thus, the incidence of memory complaints is likely underestimated. Lastly, it is important to highlight the absence of information with an informant (cognitive and functionality) and data about ancillary tests/neuroimage in this population.

This study showed that memory complaints affected three in five adults in Southern Brazil 15 months following the beginning of the COVID-19 pandemic. Besides future epidemiological studies, strategies to mitigate the impact of the COVID-19 pandemic on cognitive functioning in these identified groups are required.
